# Dr. Girdlestone, on Tapping at the Navel

**Published:** 1807-04

**Authors:** Thomas Girdlestone

**Affiliations:** Yarmouth


### *To the Editors of the Medical and Physical Journal.*

GENTLEMEN,

THE perusal of Dr. Huggan's Letter in your very useful Journal, has tempted me to send you the following remarks on Tapping at the Navel.

Soon after the year 1792, when the 3d Volume of the Medical Memoirs, which contains Dr. Sims's recommendation of tapping at the navel, was published; one of the senior surgeons of this town had tapped one of my patients a second time.

To all appearance, the second operation was performed on the same part of the left side, as in the first tapping.—But to our great surprise, instead of water, the discharge had so much the colour of arterial blood, that the surgeon apprehended he had been so unfortunate as to wound the epigastric artery. After the bandage had been tightly placed round the patient, he continued to bleed, during the greatest part of the next day, without his seeming to be at all weakened by the loss which he had sustained. On the contrary, in fewer days than after the preceding tapping, he was able to walk about, and repeat his Bacchanalian habits.

The next time he required to be tapped, the surgeon proposed to me his performing the operation at the navel, as recommended by Dr. Sims: it was immediately put into practice, and the patient was so much pleased with it, from the little or no pain which it occasioned; and the surgeon and myself were so satisfied with the success of the operation, that it became almost the invariable mode of tapping in abdominal dropsies in this town, for these last 13 or 14 years, without any inconveniency having been experienced from it, till a few months since in the following case.

A woman in this neighbourhood had been several times tapped in this manner by a practitioner in my presence, and at each tapping, about three gallons of water were discharged in less than one hour. But the last time this operator repeated on her the tapping, I perceived issuing from the side of the navel, a slender stream of blood, which did not cease to flow after the water had drained away. In spite of all the efforts of the practitioner, the bleeding continued till within a few hours of her death, which did not take place till five or six days after the operation had been performed.

Had this hæmorrhage taken place during the first operation on this woman, the vessels of the umbilical chord might have been considered, as having remained in a pervious state from infancy. But as this very uncommon variety in the adult subject was not her case, and as the circular part of the navel was cut by one of the shoulders of the lancet, I should account for this bleeding from the lancet having been introduced too far into the navel. The woman was in a state both from poverty and disease, which which made it very unlikely that she should have survived many operations, had not the bleeding hastened her termination.

That this operation is less painful than any other mode of tapping I am fully convinced: and though it cannot be expected, that they who have never seen the operation performed, will suppose the water to be as perfectly discharged, as when the tapping is made in more depending parts, yet I believe that the water is seldom more completely drawn off than at the navel. And, notwithstanding the disaster which I have mentioned, I am of opinion that no vessel of any consequence will ever be wounded, if the operator takes care only to puncture the middle of the distended part of the navel, with as light a hand as if he were penetrating a common bladder. This superficial puncture has been fully sufficient for the discharge of the water, in every case where I have seen this operation performed. Nor did I ever know any of those rigors, and other symptoms of peritonæal inflammation after this operation, which sometimes follow tapping in other parts of the abdomen. But if in any instance this superficial puncture should fail to let out the water, the recollection of this woman's case would lead one to propose the immediate recourse to tapping on the linea alba, rather than to hazard a deeper opening at the navel.

I am, &c.

THOMAS GIRDLESTONE, M. D.

*Yarmouth, March 5, 1807.*

